# Therapeutic potential of tyrosine-protein kinase MET in osteosarcoma

**DOI:** 10.3389/fmolb.2024.1367331

**Published:** 2024-03-26

**Authors:** Ming Zeng, Can Liu, Haoli Gong, Zhongwen Tang, Jie Wen, Sisi Wang, Sheng Xiao

**Affiliations:** ^1^ Department of Pediatric Orthopedics, Hunan Provincial People’s Hospital, The First Affiliated Hospital of Hunan Normal University, Changsha, Hunan, China; ^2^ Department of Anatomy, Hunan Normal University School of Medicine, Changsha, Hunan, China; ^3^ Department of Oncology, The Second Xiangya Hospital of Central South University, Changsha, Hunan, China

**Keywords:** osteosarcoma, tyrosine-protein kinase, met, targeted therapy, therapeutic potential

## Abstract

Osteosarcoma, the most prevalent primary bone tumor in children and young adults, can often be successfully treated with standard chemotherapy and surgery when diagnosed at an early stage. However, patients presenting with metastases face significant challenges in achieving a cure. Despite advancements in classical therapies over the past few decades, clinical outcomes for osteosarcoma have not substantially improved. Recently, there has been increased understanding of the biology of osteosarcoma, leading to the identification of new therapeutic targets. One such target is MET, a tyrosine kinase receptor for Hepatocyte Growth Factor (HGF) encoded by the MET gene. *In vitro* and *in vivo* studies have demonstrated that the HGF/MET pathway plays a crucial role in cancer growth, invasion, metastasis, and drug resistance across various cancers. Clinical trials targeting this pathway are already underway for lung cancer and hepatocellular carcinoma. Moreover, MET has also been implicated in promoting osteosarcoma progression. This review summarizes 3 decades’ worth of research on MET’s involvement in osteosarcoma and further explores its potential as a therapeutic target for patients with this disease.

## Introduction

Osteosarcoma (OS) mainly affects children and is characterized by abnormal formation of bone tissue and spindle cells. It originates from primitive bone-forming cells in the mesenchyme. Typically, the tumor forms near the growth plate in the long bones of the limbs, such as the shinbone, upper arm bone, and thigh bone. Occasionally, it may occur in the pelvis, jaw, or skull. Histologically, OS comprises malignant osteoblasts that produce immature bone and osteoid tissue. It can be classified into different subtypes including conventional, low-grade central periosteal, chondroblastic, parosteal, telangiectatic, and small cell varieties. Osteosarcomas are highly aggressive, often leading to symptoms such as severe pain, noticeable swelling, and frequent fractures (Rossi and Del Fattore, 2023). The current treatment involves a combination of surgery, chemotherapy before surgery, and chemotherapy after surgery (Yang et al., 2022). The introduction of chemotherapy has improved the 5-year survival rate of OS patients to 60%–75%. However, more than a third of patients experience relapse and/or metastasis to the lungs, resulting in a significantly worse prognosis, with a 5-year survival rate dropping to 20% (Wu et al., 2009; Allison et al., 2012).

As there has been no significant increase in the survival rate of patients with metastatic disease over recent decades (Ning et al., 2023), numerous studies have focused on understanding the molecular mechanisms underlying tumorigenesis and drug resistance in osteosarcoma. Targeted therapy, which includes immune system targets, drug delivery systems, as well as intercellular and extracellular signaling pathways of metabolism, has advanced rapidly in recent years (Shaikh et al., 2016). The discovery of MET has opened a new avenue for research and treatment of osteosarcoma. The receptor-tyrosine kinase (RTK) MET, encoded by the MET proto-oncogene, is activated solely by its high-affinity ligand HGF (also known as scatter factor). Initially identified as a product of chromosomal rearrangement induced by carcinogens in the human osteosarcoma cell line HOS, MET was formed through fusion between the translocated promoter region (TPR) locus on chromosome one and the MET sequence on chromosome 7. Consequently, this encodes a constitutively active TPR-MET protein with a molecular weight of 65 kDa.

Abnormal HGF/MET signaling is implicated in the development and metastasis of various malignant tumors, including sarcomas. While MET mutations are rare in sarcomas, overexpression of MET is common and associated with poor clinical outcomes in patients with this disease. Both wild-type and persistently activated MET overexpression have been shown to induce human primary osteoblasts into osteosarcoma (Patane et al., 2006). Molecular pathology and cancer biology data suggest that HGF/MET signaling plays a crucial role in the survival, growth, proliferation, metastasis, and drug resistance of osteosarcoma (Coltella et al., 2003; Wang et al., 2012; Kunii et al., 2015).

These findings highlight the significance of dysregulated MET signaling in sarcomas. Various strategies, including tyrosine kinase inhibitors and monoclonal antibodies targeting MET activation, are currently being developed, with some already in advanced stages of clinical trials. In this review, we explore the current biological evidence regarding MET in osteosarcoma and discuss its potential as a therapeutic target for osteosarcoma patients.

## HGF/MET signaling

**FIGURE 1:**
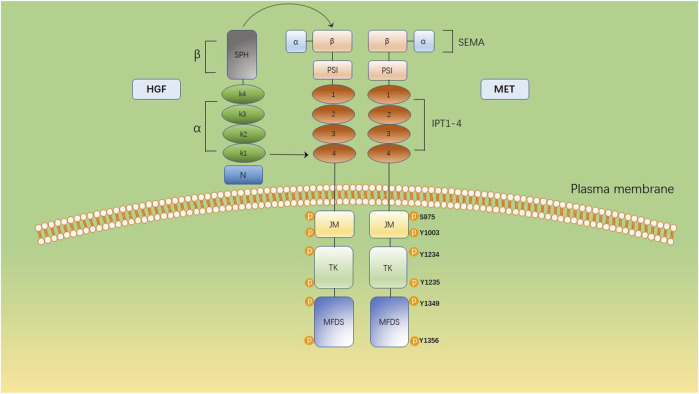
The multidomain structure of MET and its ligand, HGF. The extracellular domain comprises a SEMA domain, a plexin, semaphorin, and integrin (PSI) domain and four immunoglobulin-like IPT (IPT1–4). The intracellular domain constitutes a juxtamembrane domain (JM), containing Ser975 and Y1003 and a tyrosine kinase (TK) domain, containing Y1234 and Y1235. The C-terminal multifunctional docking site (MFDS) facilitates the recruitment of cytoplasmic signaling molecules and adaptor proteins through Y1349 and Y1356.

**FIGURE 2:**
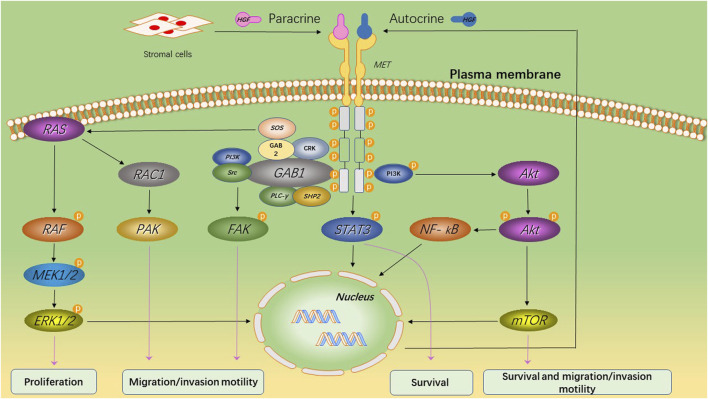
MET-induced signaling pathways and its primary biological effects. Upon binding of HGF to MET, the tyrosine kinase domain of MET initiates a cascade of phosphorylation events that lead to the activation of multiple key cell signaling pathways. This activation promotes cell survival and proliferation, while also enhancing cellular motility and invasive capacity.

HGF/MET signaling begins with the MET proto-oncogene, located on human chromosome 7q31, encoding a single 1,390 amino-acid precursor protein (Giordano et al., 1989). This precursor protein undergoes cleavage to form a mature receptor consisting of disulfide-bound α- and β-subunits (see Figure 1). The α-subunit, located extracellularly, shares homology with proteins from the semaphorin superfamily. The β-subunit comprises an extracellular domain responsible for ligand binding and an intracellular domain accountable for kinase activity and signal transduction (Gherardi et al., 2003). HGF, the sole known ligand of MET, is primarily secreted by stromal cells and activates MET on neighboring epithelial cells (Chan et al., 1991; Lokker et al., 1992; Goetsch et al., 2013). HGF binds to MET through two distinct affinity sites: the high-affinity site located in the N-terminal and first kringle regions, interacting with IPT3 and IPT4 domains in MET; and the low-affinity site situated in the serine protease homology domain, interacting with the semaphorin domain in MET (Gherardi et al., 2006; Holmes et al., 2007). Upon binding to MET, HGF induces dimerization of MET and phosphorylation of tyrosine residues Y1003, Y1234, and Y1235. It further phosphorylates tyrosine residues Y1349 and Y1356 within the multi-docking site at the C-terminus tail of the receptor (Giordano et al., 1989; Birchmeier et al., 2003). Phosphorylation of Y1349/1,356 is critical for activating downstream signaling pathways by recruiting intracellular adaptor proteins and signaling molecules that rely on src homology two domain-mediated interactions. These include SRC, phospholipase C γ (PLCγ), Shc protein (Shc), CRK adapter protein (CRK), growth-factor receptor-bound protein 2 (GRB2), GRB2-associated binding protein 1 (GAB1), as well as the p85 subunit of phosphatidylinositol three kinase (PI3K) and signal transducer activator transcription factor 3 (STAT3) (see Figure 2) (Pelicci et al., 1995; Fixman et al., 1996; Zhang et al., 2002). This interaction with the mentioned factors activates the STAT3, PI3K/Akt pathway, along with extracellular signal-regulated kinase 1/2 (JNKs)/p38 MAPK cascades, leading to nuclear factor κB(NF κB) pathway activation associated with survival, migration, and invasion processes (Rahimi et al., 1998; Maroun et al., 1999; Sipeki et al., 1999; Royal et al., 2000; Saxton et al., 2000; Kumari et al., 2005).

## Molecular alterations of HGF/MET in human OS

Several investigations have aimed to understand the genetic and functional changes of the HGF/MET pathway in human osteosarcoma, considering its crucial role in regulating cell proliferation and apoptosis in the liver (Table 1). As mentioned earlier, MET activation in human tumors can result from gene-activating mutations, amplification, or overexpression.

**TABLE 1:** MET alterations in osteosarcoma.

Author	Year	MET alterations	Findings
Ferracini et al. (1995)	1995	MET overexpression	MET was overexpressed in 60% of the osteosarcomas and 12 osteosarcoma cell lines
Scotlandi et al. (1996)	1996	MET overexpression	MET was overexpressed in the all primary (100%), local recurrences (100%), and most metastases (71%) osteosarcomas. (Western blotting)
			MET was overexpressed in the primary (95%), local recurrences (100%), and most metastases (82%) osteosarcomas. (Immunohistochemical analysis)
Rong et al. (1993)	1993	MET overexpression	osteosarcomas showed significant Met and HGF staining by confocal laser scan microscopy
Oda et al. (2000)	2000	MET overexpression	MET was express positive in 5 of 25 in the primary site *versus* 12 of 24 in the metastatic site (Immunohistochemical analysis)
Arihiro and Inai (2001)	2001	MET overexpression	MET was overexpressed in 71% osteosarcoma. (Immunohistochemical analysis)
Entz-Werle et al. (2007)	2007	*MET* gene amplification and deletion	quantitative polymerase chain reaction (QPCR) targeting *MET* indicate *MET* was amplified in 9% (8 of 88) and deleted in 41% (36 of 88)
Kawano et al. (2023)	2023	MET overexpression	MET was overexpressed in osteosarcoma cell (HOS, SaOS, and MG-63)

In recent years, several studies have assessed the expression of MET and HGF in osteosarcoma. One study examined frozen samples from 87 primary bone and soft tissue tumors and found that osteosarcoma exhibited the highest levels of MET/HGF expression. Among 46 human osteosarcoma cases analyzed, p145MET was overexpressed in all primary tumors, local recurrences, and the majority of metastases.

Moreover, remarkably high levels of MET/HGF receptor expression were observed in primary tumors and recurrences. However, no evidence of amplification was found when measuring the intensity of DNA bands obtained from osteosarcomas that overexpressed the MET/HGF receptor. The consistent detection of MET proto-oncogene overexpression in both primary and recurrent lesions, as well as in the majority of metastases, is significant for understanding the pathogenesis of osteosarcoma. Immunohistochemical analysis revealed MET overexpression in primary (95%), local recurrences (100%), and metastases (82%) of osteosarcomas (Scotlandi et al., 1996). Sing Rong et al. conducted a study using confocal laser scan microscopy to examine paraffin-embedded human osteosarcoma sections stained for MET and HGF/SF. The results demonstrated significant staining of MET and HGF/SF in osteosarcomas (Rong et al., 1993). Ferracini R et al. investigated MET overexpression in up to 60% of the examined osteosarcomas (Ferracini et al., 1995). In 12 osteosarcoma cell lines, the MET/HGF receptor exhibited overexpression, phosphorylation upon HGF stimulation, and full functionality. HGF was detected in two out of seven clinical specimens of osteosarcomas. Co-expression of the ligand and the receptor was observed in two clonal osteosarcoma cell lines. Constitutive phosphorylation of the MET/HGF receptor occurred in these cell lines; however, this phosphorylation could be suppressed by suramin treatment, a well-known blocker of autocrine loops. These findings suggest that activation of the MET/HGF receptor through either paracrine or autocrine mechanisms may contribute to the particularly aggressive behavior observed in osteosarcoma. The role of the MET oncogene product between primary and metastatic sites in osteosarcoma has remained controversial. Another study showed that all osteosarcoma cell lines had elevated MET expression in the cDNA array. The findings of this study confirm that the heat shock protein inhibitor 17-DMAG effectively inhibits the proliferation of osteosarcoma cells and induces apoptosis by targeting MET (Kawano et al., 2023).

In small groups comprising 25 patients, seven cases (28%) that showed negative MET reaction at the primary site exhibited immunoreactivity for MET at the metastatic site, indicating a higher incidence of MET expression compared to the primary site. Among all tumors, those positive for MET displayed significantly elevated MIB-1 LI compared to those negative for MET (negative: 20.99; positive: 27.65; *p* = .0292). The positive correlation between MET expression and proliferative activity also suggests a potential significant role played by MET expression in tumor progression (Oda et al., 2000). Another immunohistochemical study found that plasma membrane and cytoplasmic staining for MET/HGF receptor were positive in 71% of osteosarcoma cases (Arihiro and Inai, 2001).

A distinct approach was taken by Natacha Entz-Werle and colleagues (Entz-Werle et al., 2007) to investigate the role of MET activation in osteosarcoma. Since osteosarcomas are histologically characterized by malignant osteoblasts producing an osteoid component, this research team examined the genomic status of MET, TWIST, and APC genes involved in ossification processes in pediatric osteosarcomas treated with the OS94 protocol. The analysis included allelotyping, real-time quantitative polymerase chain reaction (qPCR), gene sequencing, and protein polymorphism study for MET in 91 osteosarcoma cases. No microsatellite instability was observed, but allelic imbalance was found in 52% of cases at locus 7q31 containing MET. By qPCR analysis alone, it was determined that the MET gene was normal in 50% (44 out of 88) of cases, amplified in 9% (8 out of 88), and deleted in 41% (36 out of 88). Notably, significant statistical associations were identified between relapsed tumors and abnormalities involving MET (70%, predominantly deleted; *p* = .004) as well as TWIST abnormalities (69%, predominantly deleted; *p* = .03). When comparing molecular profiles between deceased children and surviving patients, statistically significant trends indicated a higher frequency of deletions for both MET (*p* = .03) and TWIST genes (*p* = .01), along with a more frequent rearrangement involving APC gene (*p* = .03). Furthermore, aberrations related to MET serve as significant indicators for poor event-free survival, particularly when amplification is present (*p* = .015), suggesting that tumors with amplified levels perform worse than those with deletions.

## The involvement of the MET/HGF receptor in OS proliferation and invasive behavior

The involvement of the MET/HGF receptor in OS proliferation and invasive behavior has been extensively studied. Activation of the HGF/MET axis in OS cell lines has been shown to trigger downstream signaling pathways contributing to motility, mitogenesis, and morphogenesis in a cell type-specific manner. Nadia Coltella et al. investigated the impact of MET receptor activation on human OS cell lines by assessing HGF-dependent MAPK and PKB/AKT activation levels as biochemical indicators of mitogenic and invasive responses, respectively. Stimulation with HGF led to ERK1/2 phosphorylation in U-2OS, Saos-2, IOR/OS9, and IOR/OS10 OS cell lines. Phosphorylation of specific residues on ERK1/2 facilitated its translocation to the nucleus and increased enzymatic activity, evidenced by phosphorylation of its physiological substrate, the Elk-1 transcription factor at Ser 383 (Coltella et al., 2003). In U-2OS and Saos-2 cells, HGF consistently induced PKB/AKT phosphorylation, leading to subsequent enzymatic activation of its physiological substrate GSK-3α/β. A modest AKT response to HGF was also observed in MG-63 and IOR/OS10 cells. Cell cultures treated with 100 ng/mL HGF demonstrated a proliferative response dependent on HGF stimulation for all cell lines except MG-63, where the MAPK cascade was not activated.

The cell lines U-2OS and Saos-2, which exhibited HGF-induced activation of PKB/AKT, displayed migratory capability through the micropore filter of a Transwell chamber in response to recombinant HGF. Both HGF-dependent responses were reversed by the specific MET inhibitor K252a. The activation of the MET receptor by HGF is implicated in OS progression, as it confers functions facilitating metastatic diffusion and invasion of both local and peripheral tissues. Metastasis is a complex process involving multiple steps, with motility and invasion through the basement membrane being crucial stages. This study has demonstrated that HGF stimulates motility in OS cell lines where MET activation also triggers AKT activation. Furthermore, OS cells can invade collagen matrix in an HGF-dependent manner if they exhibit an AKT response to HGF.

Another study investigated the signal transduction and downregulation of MET in HGF-stimulated low and highly metastatic human OS cells (Husmann et al., 2015). The mRNA and protein expression levels of these molecules, as well as HGF-stimulated signaling and downregulation of MET, were compared between the parental low metastatic HOS and MG63 cell lines, their respective highly metastatic MNNG-HOS and 143B cell lines, along with the MG63-M6 and MG63-M8 sublines. TPR-MET expression resulting from a chromosomal rearrangement induced by treatment with the carcinogen N-methyl-N′-nitronitrosoguanidine was only observed in MNNG-HOS cells (Dean et al., 1987). Akt and Erk1/2 phosphorylation were stimulated by HGF in all examined cell lines; however, phospho-Stat3 levels remained basal. Downregulation of HGF-stimulated Akt and Erk1/2 phosphorylation occurred more rapidly in the HGF-expressing MG63-M8 cells compared to HOS cells. Degradation of activated MET primarily occurred through the proteasomal pathway, with a lesser contribution from the lysosomal pathway in these cell lines. Therefore, targeting HGF-stimulated Akt and Erk1/2 signaling pathways as well as proteasomal degradation of activated MET could be potential therapeutic strategies for OS. In both normal and tumor cells, Akt, Erk1/2, and Stat3 are known targets of MET signaling (Goetsch et al., 2013). However, only Akt and Erk1/2 were found to be phosphorylated upon HGF treatment in the examined OS cell lines. The absence of HGF-induced Stat3 phosphorylation suggests that Stat3 is not activated by MET specifically in these cell lines (Yan et al., 2015). This observation further supports the notion that the activation of signaling pathways by MET is contingent upon the specific cellular context.

The MET inhibitor effectively suppressed colony formation in all tested cell lines and specifically attenuated the motility of the MNNG cell line (Messerschmitt et al., 2008), which is consistent with the known expression of the TPR-MET oncogene. Furthermore, it was observed that the MET inhibitor not only impacted the MNNG cell line expressing TPR-MET but also inhibited colony formation in TE85 and 143B cell lines lacking TPR-MET expression (Peschard and Park, 2007). Therefore, incorporating a MET inhibitor into a chemotherapeutic regimen could potentially offer benefits for OS patients, irrespective of TPR-MET expression.

## MET as a prognostic marker in OS

Abnormal activation of MET has emerged as a crucial factor in the advancement and growth of OS. Traditionally, the tissue response to chemotherapy before surgery has been seen as the primary factor predicting the outcome of primary OS. Yet, recent studies have explored the link between MET activation and treatment effectiveness in OS. N Entz Werle et al. carried out research involving 54 patients under 20 years old with primary OS who underwent treatment as per the French Society of Pediatric Oncology OS 94 plan (Entz-Werle et al., 2003). They collected paired normal and biopsy samples, and surgical specimens were obtained from 13 cases post preoperative chemotherapy. Genomic DNA was extracted, followed by an analysis targeting microsatellites linked to chromosome 7q31 regions. The findings at the 7q31 locus and the region involving APC show a significant involvement of these areas in OS cancer development, despite the relatively low occurrence of changes at this locus (34%). However, among patients with partial remissions, relapses, or fatalities, the frequency of this allelic imbalance rose to 80%, indicating a strong link with this unfavorable prognosis subgroup (*p* = 0.04). Thus, these results have pinpointed a new area implicated in OS prognosis. The noteworthy connection between changes in chromosome 7q31 and worse prognosis underscores its prognostic importance.

These findings are consistent with prior research on MET and suggest its potential involvement in aggressive osteosarcoma forms (Ferracini et al., 1995; Oda et al., 2000). Understanding allelic imbalances at this locus could be crucial for predicting relapse in patients achieving partial or complete remission and may spur future use of kinase inhibitors in osteosarcoma therapy.

As previously mentioned, Natacha Entz-Werle et al. (Entz-Werle et al., 2007) conducted a study on the genomic status of various genes implicated in the ossification process in 91 pediatric osteosarcoma patients using the OS94 protocol. MET abnormalities, particularly MET amplification, are significant markers of poor event-free survival (EFS) and are often linked with inferior overall survival (OS). Tumors with MET amplification have a worse prognosis compared to those with deletions. Specifically, patients with MET-amplified tumors exhibit a 5-year EFS rate of only 28% and a 5-year OS rate of 53%. Additionally, the identification of MET abnormalities can further stratify the overall poor outcome group and pinpoint the subset of patients with the most adverse prognosis.

The activation patterns of receptor tyrosine kinases (RTKs) were also investigated in chemonaive fresh frozen tissues from osteosarcoma patients using a multiplex immunoassay (Chaiyawat et al., 2017). This analysis unveiled distinct RTK tyrosine phosphorylation patterns in osteosarcoma cases. Unsupervised hierarchical clustering, utilizing the Pearson uncentered correlation coefficient, classified RTKs into two groups: Group A (MET, c-Kit, VEGFR2, and HER2) and Group B (FGFR1 and PDGFRα), based on their tyrosine phosphorylation profiles. The study found that patients with inactive Group A-RTKs tended to have shorter overall survival compared to those with at least one active Group A-RTK. Additionally, the percentage of tumor necrosis emerged as a significant adverse prognostic factor in this investigation.

In summary, a notable correlation was observed between rearrangements at 7q31 and a poorer prognosis. MET has demonstrated its efficacy as a diagnostic marker in osteosarcoma.

## The relationship between MET activation and chemosensitivity

An analysis of survival indicated that OS patients with inactive MET tended to have a worse prognosis. Consequently, a further investigation delved into the link between MET activation status and both resistance to chemotherapy and metastasis (Chaiyawat et al., 2017). Among patients who underwent the same chemotherapy regimen consisting of doxorubicin and cisplatin (n = 10), a skilled pathologist assessed their tumor necrosis levels post-treatment. The results revealed that OS patients with active MET displayed higher sensitivity to chemotherapy compared to those in the inactive group, as evidenced by significantly reduced levels of tumor necrosis in the latter. However, no association was observed between MET activation status and metastasis.

Furthermore, primary osteosarcoma cells were exposed to varying concentrations of doxorubicin or cisplatin, followed by evaluation of cell viability using the MTT assay. The findings indicated that most primary OS cells expressing active MET exhibited heightened sensitivity to both doxorubicin and cisplatin treatments compared to those lacking MET activation. Additionally, a positive correlation was noted between the number of activated MET and increased sensitivity to doxorubicin and cisplatin in primary OS cells.

## The HGF/MET axis as a therapeutic target in OS

The occurrence and progression of malignant osteosarcoma are influenced by genetic factors and pathological changes. Angiogenesis plays a crucial role in the proliferation, migration, invasion, and metastasis of OS. Mounting evidence suggests that inhibiting angiogenesis could be a viable approach for OS treatment (Peng et al., 2016; Wang et al., 2017). Various angiogenesis inhibitors, such as sorafenib, sunitinib, and cediranib, have been employed to manage advanced OS (Coventon, 2017). However, recent studies indicate that these inhibitors may induce tumor adaptation and disease progression, fostering the development of highly invasive and metastatic tumors (Ebos et al., 2009; Paez-Ribes et al., 2009). Consequently, novel agents capable of concurrently inhibiting the VEGF signaling pathway along with other pathways involved in tumor invasion and metastasis hold promise as effective therapies for OS patients.

Anlotinib is an orally available, highly potent, multi-targeted tyrosine kinase inhibitor (TKI) that effectively impedes the phosphorylation of VEGFR2 and demonstrates activity against MET, platelet-derived growth factor receptors α/β (PDGFR α/β), c-Kit, Aurora-B, Ret, c-FMS, and discoidin domain receptor 1 (DDR1) (Sun et al., 2016; Wang et al., 2016). The results from phase II and III clinical trials have showcased the promising clinical efficacy of anlotinib in various solid tumors, including non-small cell lung cancer, hepatocellular carcinoma, renal carcinoma, gastric cancer, and soft tissue sarcoma. Anlotinib garnered approval from the CFDA for treating NSCLC in 2018.

Moreover, ongoing phase II trials have revealed significant activity of anlotinib against a broad spectrum of soft tissue sarcomas. The anti-tumor activity and underlying mechanism of anlotinib in osteosarcoma were explored (Wang et al., 2019). Various *in vitro* and *in vivo* models were employed to evaluate the effectiveness of anlotinib in terms of anti-proliferative, anti-angiogenic, and anti-metastatic properties. Anlotinib effectively suppresses tumor growth, enhances chemo-sensitivity, and inhibits migration and invasion in OS cells. Additionally, the molecular mechanism underlying its anti-tumor effects was elucidated using phospho-RTK antibody arrays, confirming its ability to inhibit the phosphorylation of MET and VEGFR2 along with activation of downstream signaling pathways. Furthermore, it was observed that anlotinib blocks HGF-induced cell migration and invasion, as well as VEGF-induced angiogenesis (Sun et al., 2016). Notably, in the 143B-Luc orthotopic OS model, treatment with anlotinib exhibited significant inhibition of tumor growth and lung metastasis. These preclinical findings collectively underscore the potential clinical utility of anlotinib as a novel inhibitor targeting MET and VEGFR2 to suppress tumorigenesis in OS.

The compound PHA-665752 is a powerful, selective, and ATP-competitive inhibitor of MET. In 2003, Christensen et al. found that PHA-665752 effectively stops the MET-dependent behavior in lab settings and shows anti-cancer activity in live subjects (Christensen et al., 2003). Later, Ma et al. showed that PHA-665752 works together with rapamycin to stop the growth of non-small cell lung cancer cells (Ma et al., 2005). Additionally, Puri et al. noted that PHA-665752 significantly reduces the growth of lung cancer tumors in mice and the formation of new blood vessels by more than 85% (Puri et al., 2007). This compound has demonstrated its ability to hinder the progression of OS, encourage cell death, and slow down the multiplication of human OS cells (Chen et al., 2021). Furthermore, the ERK1/2 pathway plays a critical role in mediating the anti-cancer effects of PHA-665752 in OS. This was reinforced by the discovery that LY3214996, a highly selective inhibitor of the ERK1/2 pathway, counteracted the effects of PHA-665752 in OS. Moreover, PHA-665752 effectively reduced tumor growth in a mouse model with transplanted tumors (Chen et al., 2021). Overall, MET presents a target for OS treatment, and PHA-665752 emerges as a promising option for fighting OS.

Cabozantinib (XL184) is a special inhibitor of VEGFR2 tyrosine kinase, which also effectively inhibits the MET receptor (Uitdehaag et al., 2014). It has shown anti-tumor effectiveness both in laboratory settings and in live subjects in different models of osteosarcoma and Ewing sarcoma (Houghton et al., 2015).

In collaboration with the Cancer Therapy Evaluation Program of the National Cancer Institute, the French Sarcoma Group conducted a clinical study (CABONE) comprising two phase II trials to explore the potential of cabozantinib in patients with advanced Ewing sarcomas and advanced OSs respectively. One of the trials focused on evaluating the efficacy and safety of cabozantinib in patients with advanced OS (Italiano et al., 2020). The primary eligibility criteria included age ≥12 years, ECOG Performance status ≤1, metastatic or unresectable locally advanced disease, and documented disease progression (according to RECIST v1.1) prior to study enrollment. There were no restrictions on the number of previous lines of treatment. Patients received daily oral doses of cabozantinib (60 mg for adults and 40 mg/m2 for children) until disease progression or unacceptable toxicity occurred. The primary endpoint was a combined objective response and non-progression at 6 months for overall survival (OS). A total of 90 patients were enrolled in the study (Ewing sarcoma: 45; OS: 45). The median follow-up duration for OSs was determined to be 31.1 months (95%CI: [24.4–31.7]). Among the evaluated OS cases after histological and radiological review, 42 patients (93.3%) demonstrated efficacy response, with seven patients (16.7%) showing partial response and fourteen patients (33.3%) exhibiting stable disease status. Additionally, fourteen patients with osteosarcoma (33.3%) remained free from disease progression 6 months after initiating treatment. The treatment was generally well tolerated; however, mild to moderate fatigue, diarrhea, mucositis, and liver transaminitis were commonly observed adverse events graded as grade 1 or grade 2 in severity among the participants. Among the more severe adverse events graded as grade 3 or grade 4 in severity were hypophosphatemia experienced by eight individuals (8.9%), an increase in aspartate aminotransferase levels reported by five individuals (5.6%), palmo-plantar syndrome observed in five individuals (5.6%), pneumothorax encountered by five individuals (=5), and neutropenia recorded among five individuals as well (5%). Furthermore, it is worth noting that at least one serious adverse event was reported among sixty-one participants accounting for approximately 67.8% of the total patient population. The efficacy of cabozantinib in patients with advanced osteosarcoma was significantly demonstrated, suggesting its potential as a novel therapeutic option in this clinical context.

Abnormal activation of MET is commonly observed in osteosarcoma patients, and MET inhibitors are acknowledged for their potential to suppress tumors. However, OS often becomes resistant to MET inhibitors, which poses a significant challenge that needs addressing.

The small-molecule inhibitor crizotinib, also known as PF02341066, has shown strong selectivity as an ATP-competitive inhibitor of MET kinase in various human tumors. It has undergone phase II/III clinical trials for treating non-small-cell lung cancer (De Mello et al., 2020). *In vitro* studies have demonstrated that specific inhibition of highly phosphorylated MET-expressing OS cells by PF02341066 effectively triggers cell death. However, the inhibitory effectiveness of PF02341066 is compromised *in vivo* due to interference from the vascular niche. A study revealed that crizotinib displayed concentration-dependent inhibition of OS cell line proliferation. Additionally, it significantly induced apoptosis. Furthermore, crizotinib caused an increase in G0/G1 phase cells and a decrease in S phase cells compared to the control group. Moreover, crizotinib effectively suppressed migration and invasion of osteosarcoma cells while decreasing the expression of MET/Gab1/STAT5 (Jia et al., 2023). OS cells located near microvessels or exhibiting vascular mimicry suppress both MET expression and phosphorylation.

Furthermore, acquired drug resistance in OS cells is linked to the activation of VEGFR2 (vascular endothelial growth factor receptor 2). Dual targeting of both MET and VEGFR2 has proven effective in reducing tumor size in a xenograft model. Combining targeted therapy against MET with VEGFR2 inhibition may offer significant benefits towards achieving an optimal therapeutic outcome for patients with OS (Tang et al., 2022). Together, these findings underscore the critical role played by tumor heterogeneity and the microenvironment in determining drug response while uncovering the molecular mechanisms behind acquired drug resistance to targeted therapy against MET.

## Conclusion

In conclusion, MET is commonly overexpressed in OS tissue and is often linked with a poor prognosis. Current *in vitro* experiments have verified that MET activation can boost the proliferation and invasion abilities of OS cells. Numerous MET inhibitors have shown notable efficacy in inhibiting tumor growth in both preclinical and clinical settings. Interestingly, patients with elevated MET expression levels tend to display heightened sensitivity to chemotherapy drugs. Histological and genomic investigations have identified MET as a promising target for assessing the prognosis and treatment of OS. However, further characterization is necessary to delve into the therapeutic implications of inhibiting MET in OS, utilizing more pertinent preclinical models and specifically tailored clinical trials.
